# Clinical Implications and Impact of Discovery of the Thyroid Hormone Receptor on Integrin αvβ3–A Review

**DOI:** 10.3389/fendo.2019.00565

**Published:** 2019-08-23

**Authors:** Aleck Hercbergs

**Affiliations:** Department of Radiation Oncology, Cleveland Clinic, Cleveland, OH, United States

**Keywords:** cancer, euthyroid hypothyroxinemia, hypothyroidism, integrin αvβ3, L-thyroxine, thyroid hormone receptor

## Abstract

Hypothyroidism has been reported to improve survival in cancer patients but only recently has the putative mechanism been identified as a receptor for thyroxine and tri-iodothyronine on integrin αvβ3. Recognition of divergence of action of the pro-oncogenic L-thyroxine (T4) from pro-metabolic 3,5,3′-triiodo-L-thyronine (T3) has enabled clinical implementation whereby exogenous T3 may replace exogenous (or endogenous) T4 to maintain clinical euthyroid hypothyroxinemia that results in significantly better survival in advanced cancer patients without the morbidity of clinical hypothyroidism.

## Introduction

In recent years there have been reports that blood thyroxine depletion in individuals with advanced solid cancers–e.g., glioblastoma, high grade soft tissue sarcoma, was associated with regression of advanced tumors. Exogenous 3,5,3′-triiodo-L-thyronine (L-T3) was used to maintain metabolic euthyroidism (1, 2).

How did this approach come about?

The 1993 report of spontaneous remission (3) and 5-year survival of a patient with metastatic non-small cell lung cancer inspired this novel line of research, which has evolved from early experimental studies to the clinic, wherein proactive medically induced thyroid suppression was investigated in the treatment of failed high grade brain tumors (glioblastoma multiforme) (4). Significant prolongation of life (from 4 to 10 months median survival) was associated with a drop of >40% of circulating free thyroxine (T4) levels. Tumor regression was also observed in patients. There was short survival in all of the 50% of patients who did not experience free thyroxine depletion. The delay in thyroid hormone decrease remained an obstacle in extended implementation of this approach until the discovery by Davis and Mousa et al. of a newly identified cell surface receptor for thyroid hormone (both analogs T4 and T3) on integrin αvβ3 expressed on cancer cell membranes and actively dividing vascular endothelium (5).

This contribution is not intended as a comprehensive review of the literature of thyroid hormones and cancer but is focused on relevance of thyroxine impact on cancer growth and biology, now understood to be mediated through the cell surface integrin αvβ3 receptor.

## Thyroid Hormone in Physiology and Cancer Biology

### T4 and T3–A Pivotal Difference and Roles

The use of exogenous T3 as a substitute for T4 as thyroid hormone replacement has enabled withdrawal of the potent pro-oncogenic hormone T4 in cancer patients without resulting in clinical hypothyroidism (1).

In the absence of T4, cancer cell mitogenesis and proliferation does not occur. Absence of T3 impacts severely mitogenesis and metabolism. The pivotal role of thyroid hormones thyroxine T4 and T3 in cancer growth and biology and the impact on emerging clinical practice has become understood following identification of integrin αvβ3 and the interaction with and binding differences between T3 and T4 (1). T4 is the principal secretory product of the thyroid gland. It serves as a prohormone for T3 in the latter's intracellular functions (1). The reported literature refers solely to blood thyroid hormone levels individually, e.g., FT4 or FT3 and/or TSH or under the “hypothyroid” label.

T3-directed gene expression in cells requires primary interactions between T3 and its nuclear receptor proteins (TRs) (1, 2). T3 also regulates mitochondrial respiration (3). Because its biological half-life is significantly longer than that of T3 (6), T4 is the most commonly prescribed form of thyroid hormone replacement for clinical hypothyroidism and for suppression of endogenous pituitary thyrotropin (TSH) (1).

It is becoming accepted that cancer depends on both analogs to exist, survive, proliferate, and grow into tumors. Thyroxine is the sole endogenous and pleiotropic pro-oncogenic hormone that abets cancer, acting as a ligand for the membrane expressed receptor on integrin αvβ3 (7).

Thyroid hormone is a pivotal crucial pro-growth pro-oncogenic hormone for most if not all malignant tumors and the crucial interaction of T4 is with the integrin αvβ3. Thyroid hormone drives and is pro-proliferation and pro-angiogenic for cancer (7–9).

It will become evident how this discovery has led to a new and novel paradigm in understanding and management of solid cancers.

Thyroxine is the more potent pro-oncogenic thyroid hormone analog and is the pro-hormone for T3, which is the dominant pro-metabolic thyroid hormone (10, 11). T3 and T4 bind to the plasma membrane protein integrin αvβ3, which mediates the signal across the membrane (5). T4 is active at this receptor (7, 11). *In vitro* evidence indicates that T3, bound with a lower affinity by αvβ3 (7), is of low activity at physiological levels at the receptor.

Integrin αvβ3 contains two thyroid hormone binding sites, S1 and S2, which activate different downstream pathways (12): S1 binds T3 and activates the PI3K/AKT-pathway whereas S2 binds T4 and, *with lower affinity*, T3, and activates PI3K/AKT-pathway and MAPK-pathway. At physiological concentrations, T4 (and not T3) is the principal ligand at S2. Thyroid hormone action at αvβ3 is inhibited by the deaminated and decarboxylated T4 derivative tetrac (3,3,5′,5′-tetraiodothyroacetic acid) (5). Blocking the thyroid hormone receptor on the integrin (equivalent to total thyroxine depletion) downregulates multiple pro-oncogenic genes and upregulates pro-apoptotic genes (12). The consequences of tetrac on human xenograft growth and a triple negative breast cancer gene expression are profoundly anti-oncogenic (12, 13).

Thyroid hormone is therefore a high priority molecule promoting interaction with tumor cell membranes and is not tumor specific. The pursuit of strategies to implement “precision” medicine is clearly irrelevant here.

### Genomic vs. Non-genomic Actions of Thyroid Hormone

**Figure 1:**
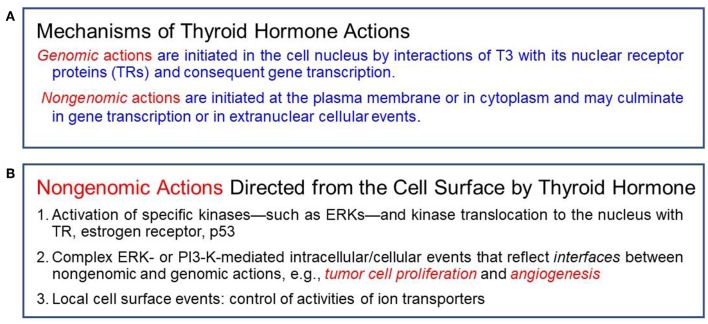
**(A)** Actions of thyroid hormone are genomic or non-genomic in mechanism. **(B)** Specific non-genomic actions.

**Figure 2:**
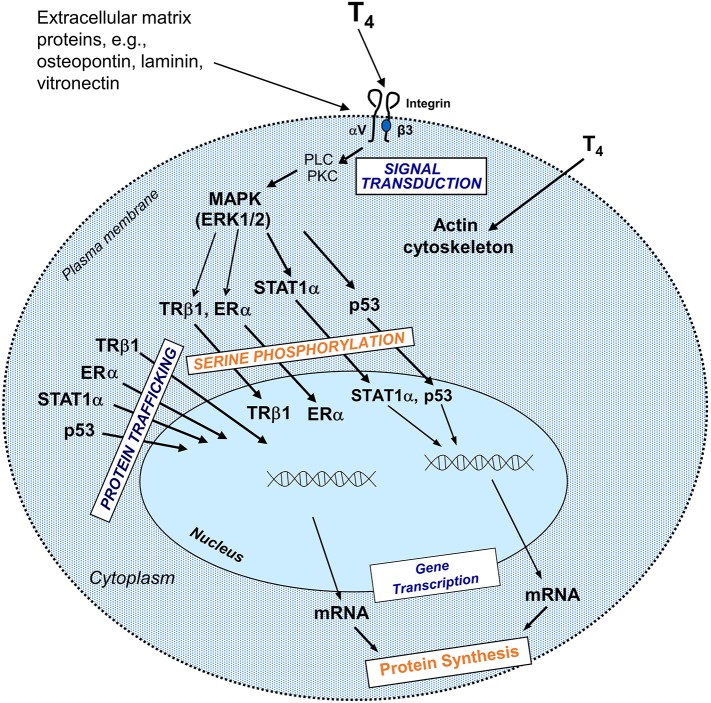
Non-genomic actions of the hormone that begin at integrin αvβ3 include regulation of intracellular trafficking of specific proteins to the nucleus and serine phosphorylation of some of these proteins in the course of nuclear entry. Directed to the nucleus from the cytoplasm, some of these proteins might be involved in modulation of transcription of specific genes and in cell proliferation. These pathways depend on activation of phospholipase C (PLC), protein kinase C (PKC), mitogen activated protein kinase (MAPK)1, and MAPK2. T4 non-genomically rapidly activates actin polymerization in hypothyroid astocytes and osteoblastic cells (15). Reprinted with permission from Hercbergs et al. (16).

Classical effects of thyroid hormones are initiated when T3 binds to its TRs that interact with specific responding elements (TREs). Thyroid hormones can also elicit their actions by a non-classical mechanism without direct gene transcription regulation by nuclear TRs (Figure 1). These non-genomic actions indirectly modulate gene transcription by activating intracellular pathways and other transcription factors (10, 14). Many of the non-genomic actions are via a receptor expressed on a membrane integrin for thyroxine and T3. The availability of T4 is the crucial step for activation of the integrin and consequent trans-membrane signaling into the cell (Figure 2). Total withdrawal or depletion of T4 will arrest this entire process with consequences for the viability of the tumor and/or vascular cell (11).

## Thyroid Hormone, Cancer, and the Integrin αvβ3-Expressed Thyroid Hormone Receptor

### T4 as a Hormone

Observations of thyroid hormone's impact on cancer prognosis predate the discovery of the integrin αvβ3 and thyroid hormone receptor on cancer cells and membranes and vascular endothelium. These studies are summarized in Table 1.

**Table 1:** Cancer outcomes across a spectrum of thyroid functions.

**Thyroid function**	**Type of research**	**No. of cases**	**Cancer type/disease**	**Clinical outcome**	**References**
Spontaneous hyperthyroid	Prospective population study	29,691	Several malignancies	Significantly higher hazard ratios for lung and prostate cancer vs. significantly lower for HT	(17)
	Case-control	532	Pancreas	Increased risk with prior hypothyroidism	(18)
	Case-control	26, 22 matched controls	Breast	Subclinical hyperthyroidism associated with more frequent cancers	(19)
Spontaneous hypothyroid	Case report	1	NSCLC, metastatic	'Spontaneous' CR following myxedema coma	(20)
	Series	28	Various solid tumors	100% response (CR and PR) rate to radiation therapy in chemically HT pts	(21)
Primary hypothyroidism-Thyroid hormone supplemented	Population-based	1,136, 1,088 controls	Breast, primary	Less aggressive disease in HT group, fewer metastases, 7 years older age at onset, smaller tumors	(22)
	Comparative study	280	Breast, all stages	5 years older for HT	(23)
	Comparative study	68, 91 matched controls	Breast, all stages	6 years older, smaller tumors, lower stage, lower S phase for HT	(24)
	Comparative study	85, 85 matched controls	Lung, all stages	4.3 years older, longer survival for HT	(25)
	Comparative study	247, 234 matched controls	RCC, all stages	Greater use of TH in RCC pts	(26)
	Case report	1	Breast	Apparent tumor stimulation with TH	(27)
	Case report/review	1	NSCLC	Apparent tumor stimulation with TH	(20)
	Case report	1	Anaplastic thyroid	Apparent tumor stimulation with TH, CR while clinically HT, 10-year survival	(28)
	Series	5	Pancreas, CRC	Long-term survival while on lower dose; TH/TH discontinued	(29)
	Series	176	Breast	Pts taking TH before diagnosis had greater relapse rate, larger tumors	(30)
Hypothyroid –[iatrogenic] 2° to XRT/CHEMORX/SURG/Biologics	Retrospective	54	RCC treated with sunitinib	Pts becoming HT with sunitinib and treated with TH seemed to have worse outcome	(31)
	Retrospective	155, with 59 developing HT	HNSCC	Pts developing HT seemed to have better survival	(32)
	Population-based	5,916 (age >65)	HN (excluding thyroid, larynx, prior HT)	Longer survival in those developing HT	(33)
	Phase II, subset analysis	34	RCC, melanoma treated with IL-2/LAK cells	Higher responses with development of HT	(34)
	Phase II, subset analysis	16	RCC, metastatic, treated with IL-2/LAK cells	Development of HT correlated with better response rate	(35)
Interventional hypothyroxinemia	Phase I-II	36	Recurrent, high-grade gliomas made HT with PTU	Early-onset HT associated with improved survival	(4, 36)
	Phase II	20	Recurrent, high-grade gliomas made HT with PTU	HT associated with improved survival	(37)
Recurrent disease following [re-] initiation of L-thyroxine in HT pts	Case reports	4	Breast	7/9 women given TH after mastectomy developed recurrence, 4 of which were late	(30)
	Case report	1	Breast	Rapid progression, death after re-starting TH, 3+ years after being in CR	(27)

*CR, complete response; CRC, colorectal cancer; HN, head and neck; HNSCC, head and neck squamous cell carcinoma; HT, hypothyroidism; IL-2, interleukin 2; LAK, lymphokine-activated killer; NSCLC, non-small cell lung cancer; PR, partial response; pts, patients; PTU, propylthiouracil; RCC, renal cell carcinoma; TH, thyroid hormone; XRT, radiation therapy. Reprinted with permission from Hercbergs et al. (38)*.

The discovery of the receptor and activation by thyroxine and significantly less by T3 has led to successful clinical translation and implementation in the treatment of compassionate care cancer patients by simple substitution of exogenous T3 for exogenous L-thyroxine in hypothyroxinemia. Discovery of the thyroid hormone receptor on integrin αvβ3 advances the understanding of the interaction and relationship to cancer with ambient thyroid hormone levels (1).

### T4 and Cancer Biology

T4 in physiological free hormone concentrations stimulates proliferation of cancer cells *in vitro* and in xenografts (13, 39–45). Preclinical studies of tetrac, which blocks thyroid hormone action at integrin αvβ3, have shown arrested tumor growth (7, 11) in a variety of tumor xenografts including xenografts of renal cell carcinoma (13), non-small cell lung carcinoma (46), medullary carcinoma of the thyroid (41), pancreatic carcinoma (43), and multi-drug resistant breast cancer (47).

## Clinical Translational Study to Induce Euthyroid Hypothyroxinemia

### Euthyroid Hypothyroxinemia and Divergence of Action Between T4 and T3

This is a eumetabolic state maintained in the total absence of blood thyroxine by providing exogenous T3. The individual can therefore live and perform all normal daily activities and functions as prior to removal of the source of T4 or post total thyroidectomy. Preclinical evidence (12) indicates that T3, bound with a lower affinity by αvβ3 (9), is of low activity at physiological levels at the receptor (5, 39). The clinical ramifications of these effects on cancer cells are supported by the results of induction of the state of euthyroid hypothyroxinemia in patients with advanced cancers and with normal thyroid function (1).

**Figure 3:**
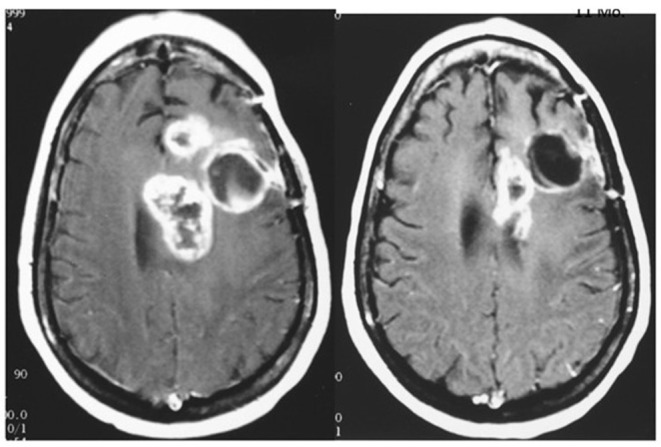
MRI of brain of a 42 year old female with recurrent glioblastoma showing significant mass reduction with free thyroxine depletion at 4 months. Left, pre-thyroxine depletion; right, 4 months later. The patient survived for 3 years. Reprinted with permission from Hercbergs et al. (4).

**Figure 4:**
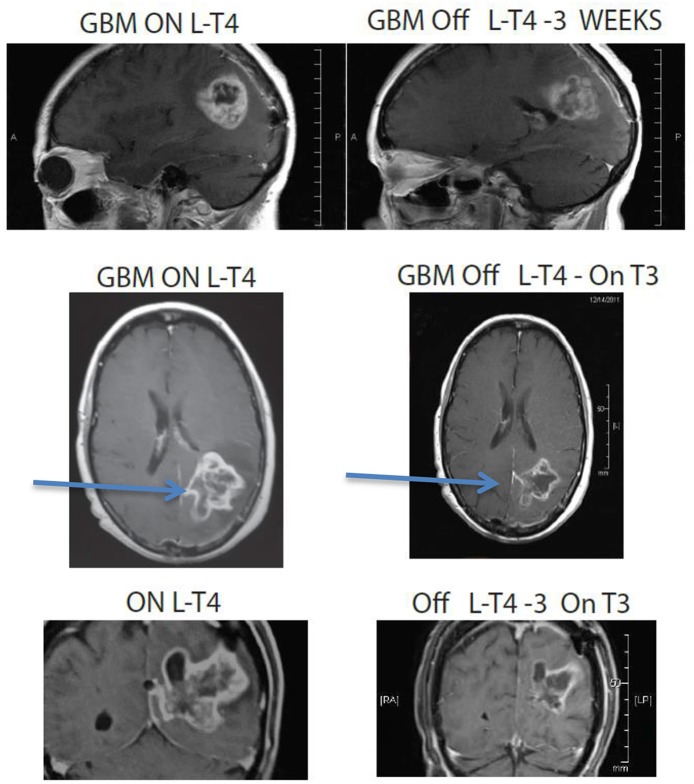
MRI images of a 67 year old female patient who was deteriorating neurologically with hemiplegia unresponsive to high dose dexamethasone. Discontinuation of L-T4 was followed within 1 week by significant clinical improvement and tumor regression. Arrows point to tumor mass, showing reduction in size in all dimensions following cessation of exogenous L-T4.

**Figure 5:**
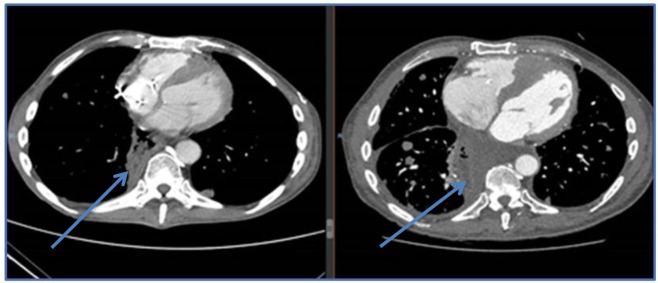
CT images of esophageal sarcoma metastatic with cardiac infiltration and cardiac failure, right image is on exogenous L-T4, left image is post L-T4 discontinuation and oral cyclophosphamide. Patient improved clinically and was discharged from intensive care.

In a compassionate-need study of terminal patients with a variety of incurable solid tumors, extended survival was observed in a majority of patients using exogenous T3 to induce and maintain hypothyroxinemia. T3 administration prevented symptomatic hypothyroidism. Low odds of survival were surmounted in 19 of 23 patients (83%) who exceeded the expected median survival of literature-reported series used as controls (1). An additional approach is to terminate exogenous T4 supplementation to allow the T4 level to decline and supplement with T3 titrated individually to the patient's functional needs. Examples of the outcomes of this strategy on advanced disease (glioblastoma and sarcoma) are shown in Figures 3–5.

## Clinical studies

Clinical studies have shown that survival is significantly prolonged (almost 3-fold) in failed glioblastoma patients treated with propylthiouracil to inhibit thyroxine synthesis.

More information available about T4 action on lung cancer is limited, but euthyroid hypothyroxinemia appears to slow the course of and extend survival of non-small cell, small cell lung, pancreas, mesothelioma, glioblastoma, and soft tissue sarcoma (1, 2).

Going forward it is suggested and clinically pivotal that the term hypothyroxinemia be employed and not the clinically imprecise term hypothyroidism, which is misleading in the emerging era as shown in this paper.

## Discussion

The identification of the pro-oncogenic exogenous thyroxine and its replacement by the metabolically dominant T3 has had a significant impact on the palliation and survival of advanced cancer patients, who live longer. This discovery followed identification of the integrin αvβ3 thyroid hormone receptor and lower binding of T3 than T4. There is also divergence of signaling pathways between the pro-metabolic T3 and the pro-oncogenic T4 (8).

Tetrac is a specific molecular blocker of the thyroid hormone receptor on integrin αvβ3 and is anti-oncogenic. The effects of tetrac occlusion of the receptor reveal the pleiotropic pro-oncogenic unopposed T4 effect on mitogenesis, angiogenesis, and apoptosis (48).

Both T3 and T4 ligand have a receptor on the cancer cell plasma membrane and on dividing vascular endothelial cells. This receptor on integrin αvβ3 is activated to transduce mitogenic signaling to the interior of the cell. Similarly, blocking of the receptor with tetrac effectively blocks all (most) T4 and T3 signal transduction as occurs following T4 and T3 depletion. This results in regression of established tumors, inhibits angiogenesis, and potentiates ionizing radiation-induced cell death (2). Separation and divergence of the effects and functions of T4 from T3 on integrin αvβ3-expressed receptors occurs as a result of binding to the receptor. T4 is significantly more potent than T3 (48) on cancer and blood vessel cells, as noted above.

T4 depletion with use of an antithyroid drug such as methimazole eliminates circulating T4 and T3, whether the latter is derived from T4 in the peripheral circulation or directly released by the thyroid gland. This approach has been utilized in cancer patients with considerable efficacy, resulting in significant prolongation of survival and tumor regression in some patients with large tumor masses, e.g., GBM and soft tissue sarcoma (1).

A rare spontaneous regression of metastatic lung cancer has occurred whereby T4 and T3 became depleted to life threatening levels with myxedema coma (3).

It is of note that there is a declining continuum of risk for free thyroxine levels from high supraphysiological (hyperthyroidism) to frank hypothyroxinemia and to blocking of the integrin αvβ3 thyroid hormone receptor, which would equate to a zero ambient free T4.

## Future Directions

The capacity/ability to impact on cancer progression in the occult or preclinical stage with only a molecular diagnosis and identification by altering endogenous thyroid hormone (thyroxine) levels might become a minimally morbid, but effective, treatment approach to pre-emptively treat solid tumor types prior to their emergence as clinically evident disease.

## Summary

Discovery of integrin αvβ3 receptor and binding to the thyroid hormone ligand have led to the strategy of rapid T4 depletion of the pro-oncogenic stimulus of T4 both in the euthyroid and exogenous T4 supplemented individuals. A significantly faster release from T4 driven tumor growth has resulted in rapid tumor regression, with palliation in life threatening situations and better survival. The discovery of tetrac binding to the receptor promises even greater therapeutic gains. The wide range of tumor types responding to T4 depletion suggests there is potential for globally impacting on cancer from the very early to late stages. It is rational to consider application of this approach to much earlier states of cancer and thereby prolong survival significantly.

## Conclusion

Identification of the integrin αvβ3 receptor pathway promises a paradigm changing approach to manage and treat cancer more effectively than currently available modalities.

## Author Contributions

The author confirms being the sole contributor of this work and has approved it for publication.

### Conflict of Interest Statement

The author declares that the research was conducted in the absence of any commercial or financial relationships that could be construed as a potential conflict of interest. The handling editor declared a past co-authorship with the author.
